# Assemblage structure and spatial diversity patterns of kelp forest-associated fishes in Southern Patagonia

**DOI:** 10.1371/journal.pone.0257662

**Published:** 2021-09-20

**Authors:** Mathias Hüne, Alan M. Friedlander, Enric Ballesteros, Jennifer E. Caselle, Enric Sala

**Affiliations:** 1 Centro de Investigación para la Conservación de los Ecosistemas Australes (ICEA), Punta Arenas, Chile; 2 Pristine Seas, National Geographic Society, Washington, DC, United States of America; 3 Hawaiʿi Institute of Marine Biology, University of Hawaiʿi, Kāneʻohe, Hawaiʿi, United States of America; 4 Centre d’Estudis Avançats de Blanes-CSIC, Blanes, Girona, Spain; 5 Marine Science Institute, University of California Santa Barbara, Santa Barbara, California, United States of America; University of Sydney, AUSTRALIA

## Abstract

Knowledge of the ecology of the fish fauna associated with kelp (primarily *Macrocystis pyrifera*) forests in Southern Patagonia is scarce, especially in how abiotic and biotic variables influence their structure, diversity, and distribution. This information is important for the management and conservation of this unique ecosystem, which has minimal anthropogenic impacts at present. We analyzed data from 122 quantitative underwater transects conducted within kelp forests at 61 stations from Chile’s southern Patagonian fjords to the Cape Horn and Diego Ramirez archipelagos and the southern tip of Argentina, including the Mitre Peninsula and Isla de los Estados. In total, 25 fish species belonging to 13 families were observed. Multivariate analysis indicated that there are significant differences in fish assemblage structure among locations and wave exposures, which was driven primarily by *Patagonotothen sima* and *Paranotothenia magellanica*, which occurred on exposed and semi-exposed stations. *P*. *cornucola* was mainly distributed across sheltered stations of the Kawésqar National Park. Temperature, salinity, depth, and kelp density influenced fish assemblage structure, with the highest diversity in areas with the lowest temperature and greater depth at Isla de los Estados. In contrast, species richness, diversity, abundance, and biomass were all lower in areas with high density of the understory kelp *Lessonia* spp., which might be driven by the absence of *P*. *tessellata*, *P*. *squamiceps* and *P*. *cornucola*, the most important species in terms of occurrence, abundance, and biomass. Our study provides the first broad-scale description of the fish assemblages associated with kelp forests along the southern cone of South America based on non-invasive visual transects, improving our knowledge of the distribution of fish assemblages across several environmental conditions in this vast and little-studied area.

## Introduction

The marine ecosystems of Southern Patagonia are amongst the least impacted on the planet. The region has a high degree of geomorphological complexity, with archipelagos, peninsulas, gulfs, channels, and fjords, which have been shaped by ice expansion and contraction during the Quaternary glacial period, giving this region a high diversity and heterogeneity of nearshore habitats [1–3]. Habitat heterogeneity is also influenced by freshwater discharge from the melting of four large ice fields (Southern Patagonia, Muñoz-Gamero Peninsula, Santa Ines Island, and Cordillera Darwin), which gives this region high environmental variability, with a strong salinity gradient between fjords (low salinity) and islands exposed to oceanic conditions (high salinity water) [4–6]. Oceanographic factors related to the confluence of water masses from the Pacific, Atlantic, and Southern oceans that mix through the Strait of Magellan and Beagle Channel result in highly diverse marine communities with species of temperate and sub-Antarctic distributions [7, 8].

The preference for certain habitat characteristics and physical conditions defines the spatial distribution of species assemblages [9]. For nearshore fishes, abiotic conditions (e.g., degree of wave exposure, bottom type, temperature, and depth) as well as biogeographic, energetic, and anthropogenic factors affect assemblage structure, diversity, abundance, and biomass on different spatial scales [10–14]. Kelp acts as the major biotic habitat-former, influencing fish assemblage structure [15, 16]. These macroalgae-forming habitats typically have high biodiversity and high production rates [17]. Fishes benefit from the three-dimensional structure of the kelp forests by providing refuge/shelter from predators and by supporting rich invertebrate communities, which provide a food source for kelp-associated fishes [18–20]. Several fish species use these kelp forests for recruitment and nursery habitat [21]. In Southern Patagonia, the giant kelp (*Macrocystis pyrifera*) is a dominant component of these forests [22], which inhabit different types of substrates across wide depth ranges and diverse environmental conditions [21–24]. In many locations of this region, the large brown seaweed *Lessonia* spp. forms dense understories within the *Macrocystis* canopy [6, 25].

More than 120 fish species have been recorded in the shallow waters (< 20 m) of the southern cone of South America [26, 27]. Thirty-five of these fish species have been recorded in association with giant kelp [6, 28–33]. Most of the fish species associated with giant kelp within this region are strongly associated with the benthos due to their lack of a swim bladder and as a result have low buoyancy and mobility [30, 34]. Many of these fish species have been poorly studied, and as a consequence there is a lack of ecological and even taxonomic information about several fish species associated with kelp forests in this region. Recent studies have helped to fill some of these knowledge gaps by characterizing fish assemblages in kelp forests across diverse environmental conditions [6, 32, 33]. However, no previous studies in this region have considered how abiotic and biotic variables influence fish species distribution, diversity, and assemblages associated with kelp forests at a large spatial scale.

The data used here were collected during the austral summers (February-March) of 2017, 2018 and 2020 as part of the National Geographic Pristine Seas Program. We characterized the fish fauna associated with *Macrocystis pyrifera* across the nearshore of: (1) Kawésqar National Park (KNP), which is characterized by channel and fjord ecosystems [33]; (2) Isla de los Estados (IE) and Mitre Peninsula (MP) at the easternmost extent of Tierra de Fuego, Argentina [32]; and (3) the Cape Horn (CP) and Diego Ramírez (DR) archipelagos, with the world’s southernmost kelp forests situated at the tip of South America [6]. The data set from this survey series is the most spatially extensive for the shallow fish assemblages of the southern cone of South America, providing for a study of the distribution of fish assemblages from multiple environmental conditions across this vast and little-studied region.

The objectives of this study were to: (1) assess the spatial patterns in kelp forest fish assemblage structure, characterizing, and comparing the shallow fish assemblages of KNP, IE, MP, CH, and DR, (2) determine which environmental parameters influence their diversity, abundance, and biomass, and (3) provide a baseline of the spatial distribution of fish assemblages for this remote region to which future changes (e.g., shifts in fish species distribution associated with climate change) can be assessed.

## Material and methods

### Ethics statement

Data were collected by all authors in a collaborative effort. Non-invasive research was conducted, which included photographs, and visual estimates described in the methods below. The Republic of Argentina and Chile granted all necessary permissions to conduct this research. No vertebrate sampling was conducted and therefore no approval was required by any Animal Care and Use Committee. Our data are publicly available at Data Dryad: doi: 10.5061/dryad.jf36b; 10.5061/dryad.6djh9w0xd; 10.5061/dryad.f7m0cfxvj.

### Study area and data collection

**Fig 1:**
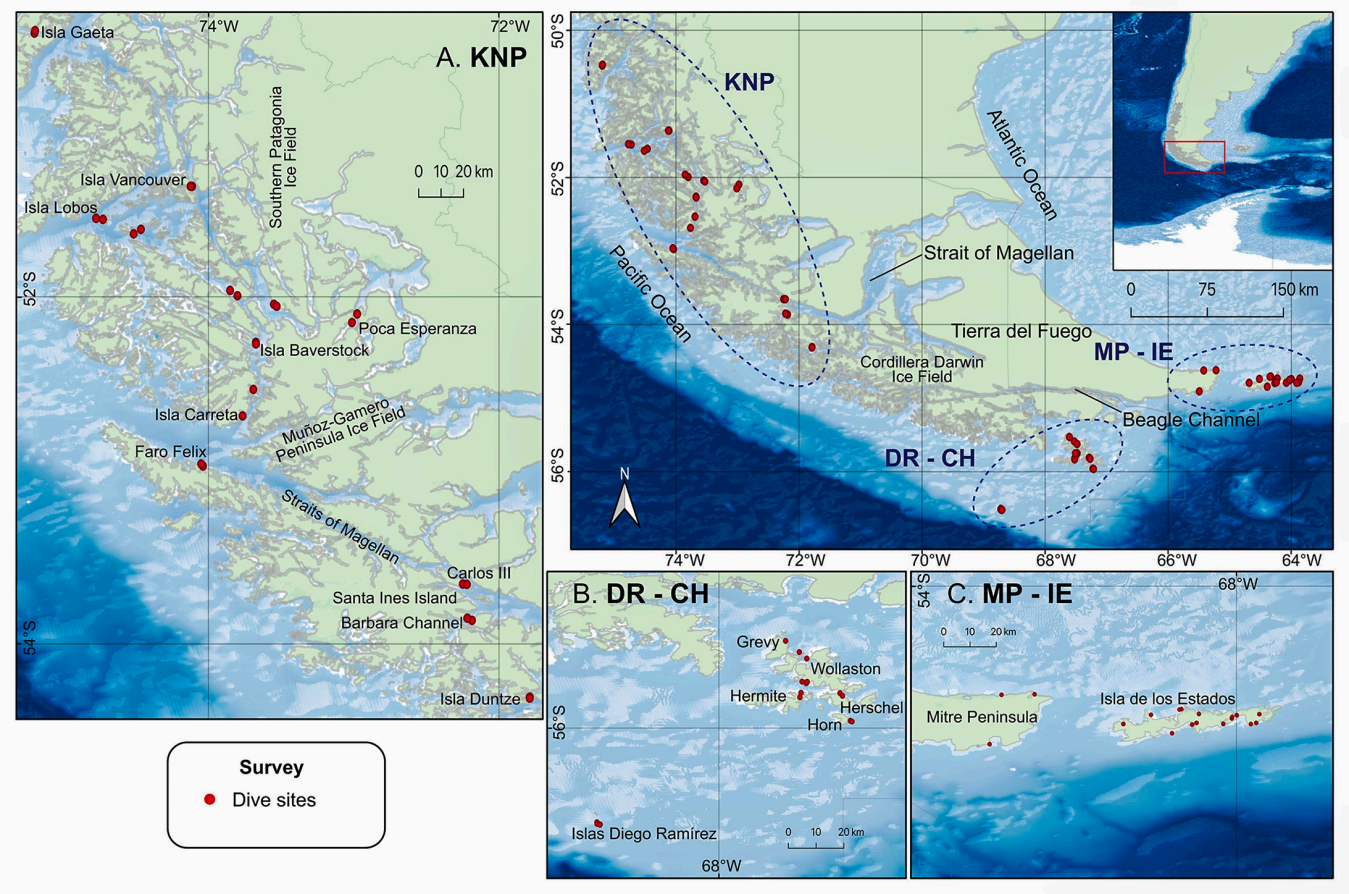
Sampling stations in Southern Patagonia. A. KNP = Kawésqar National Park, B. DR = Diego Ramírez and CH = Cape Horn Archipelago, C. MP = Mitre Peninsula and IE = Isla de los Estados. Basemap derived from GEBCO Compilation Group (2020) GEBCO 2020 Grid (doi:10.5285/a29c5465-b138-234de053-6c86abc040b9). Processing and assembly of the Global Self-consistent, Hierarchical, High-resolution Geography Database for shoreline data from [35].

Surveys were conducted within shallow forests of giant kelp, *Macrocystis pyrifera* (4–18 m depth), along approximately 800 km of the southern coast of South America between Gaeta Island (50.48^o^S 75.19^o^W) to the north and DR (56.5^o^S 68.70 ^o^W) to the south, including IE (54.7^o^S 64.5^o^W) in the southeast (Fig 1). All fish data were collected during the austral summers (February-March) of 2017, 2018, and 2020 (for more details, see [6, 33, 34]). We surveyed 61 stations (N = 122 25-m transects) in the study area, which were aggregated into five locations: CH (n = 12), DR (n = 4), IE (n = 15), MP (n = 3), and along the KNP (n = 27) (Fig 1).

### Survey method

All surveys were conducted using SCUBA along two, 25-m transects at each sampling location. Transects were conducted parallel to the shore, towards the lower edge of the kelp zone. At each survey station (N = 2 transects), one diver counted and sized all fishes within 1 m of either side of the transect line (50 m^2^). The transect area extended from the benthos to the surface, or as far as visibility allowed, including species associated with the kelp canopy and water column. Since most fish species are benthic and cryptic, transects were performed at a uniformly slow swimming speed of 2 m min^-1^ [36]. Total fish lengths were estimated to the nearest cm. Underwater photographs were taken *in situ* to assist with species identification and to document coloration and associated habitats. A second diver counted the number of kelp stipes (*Macrocystis pyrifera* and *Lessonia* spp.) within 1 m on either side of these transects (N^o^. m^-2^) and recorded the bottom type for each transect. Bottom type was classified as a single habitat type for each transect (i.e., rock, rock with sand, or rock with silt), which comprised the dominant habitat along each 25-m long transect.

Biogeographic affinities, habitat preferences, and trophic groups were assigned to all fish species using literature available for the study area [28, 30, 34, 37, 38]. Fish abundance was estimated as the number of individuals m^-2^. Biomass of individual fishes was estimated using the allometric length-weight conversion: W = aTL^b^, where parameters a and b are species-specific constants, TL is total length in cm, and W is weight in grams. Length-weight fitting parameters were obtained from FishBase [39] and for notothenioid species from Fernández et al. [40]. Salinity and temperature were recorded using a YSI model 556 handheld multiparameter instrument at KNP and an RBR Concerto multi-channel logger at CH and DR. For MP and IE, salinity and temperature measurements were obtained close to the sampling areas during February of 2015 and April of 2017, at 10 m depth [41, 42].

### Data analyses

#### General assemblage characterization

Species accumulation curves using the observed species richness (S_obs_) and the expected number of species calculated by the nonparametric estimator Chao 2 with standard deviation, were produced to assess sampling effort based on fish species occurrence on all transects [43]. These curves were constructed with 999 randomizations without replacement, using EstimateS v.9.1 [44]. Diversity was calculated using the following univariate indices: 1) total species: *S*—the number of species in each sample, 2) Margalef’s richness: *d* = (S-1)/Log(N) where S is the total number of species present and N is the total number of individuals, 3) Shannon-Weiner diversity: *H*’ = —∑p_i_ln(p_i_), where *p*_*i*_ is the proportion of all individuals counted that were of taxa *i*, 4) Pielou’s evenness: *J’* = *H*’/ln(S), where S is the total number of species present. The univariate indices were estimated with Primer v6 [45]. Comparison of diversity indices among locations were conducted using a Kruskal-Wallis rank-sum test (*X*^2^), with Dunn’s test for multiple unplanned comparisons.

#### Spatial patterns of fish assemblage and diversity

Two-factor (location and exposure) permutation-based multivariate analysis of variance (PERMANOVA) was carried out using the Bray–Curtis similarity matrix on abundance of fish species from transect data to establish to what extent locations and exposure changes impact the structure of the fish assemblages [46]. Locations (KNP, IE, MP, CH, DR) and exposures (exposed, sheltered, and semi-exposed) were treated as fixed factors with interactions. Exposures were assigned subjectively based on topography and orientation of the site to the predominant swell direction and westerly winds based on meteorological and oceanographic data [47, 48]. Shorelines directly exposed to ocean swells were classified as exposed, semi-closed channels and fjords were classified as semi-exposed, and fjords and inland bays protected from westerly winds were classified as sheltered. Permutation of residuals was under a reduced model (Sums of squares Type III–partial) with 999 permutations. Fish species abundances were ln(x+1) transformed prior to analysis. Principal Coordinate Analysis (PCO) was carried out to visualize fish assemblage structure among locations and exposures in ordination space. The primary taxa vectors driving the ordination (Pearson product-moment correlations *r* ≥ 0.5) were overlaid on the PCO plots to visualize the major taxa that explained the spatial distribution patterns observed.

Redundancy analysis (RDA) was used to determine how environmental variables influence fish assemblage structure, using abiotic factors [temperature (^o^C), salinity (ppt), depth (m)] and biotic factors (density of *M*. *pyrifera* and *Lessonia* spp.). Fish abundance data were square root-transformed prior to analyses. The significance of RDA was tested using analysis of variance (ANOVA). Variance partitioning (adjusted R^2^) was employed to quantify the relative contribution of abiotic and biotic factors.

Generalized additive models (GAMs) were used to assess the effects of habitat variables on fish diversity, abundance, and biomass. GAMs are similar to generalized linear models except that a component of each linear predictor is a sum of smooth, nonlinear functions of the predictor variables in the model. The total number of fish species (*S*), Shannon-Weiner diversity (*H*’), Numerical abundance (N^o^. m^-2^), and biomass (g m^-2^) were modeled as response variables.

Exposure, bottom type, latitude, longitude, temperature (^o^C), salinity (ppt), depth (m), and density of *M*. *pyrifera* and *Lessonia* spp. (N^o^. m^-2^) were selected as predictor variables. Variance Inflation Factor (VIF) analysis was used to test for multicollinearity. Predictor variables with VIF > 10 were removed [49]. Interaction between latitude and longitude was included in the models to assess the spatial influence. Best-fit models for each response variable were developed through a process of elimination; predictor variables were removed from the models based on their lack of significance and their collinearity with other predictors until the models with the highest deviance explained and the lowest Akaike information criterion were produced [50]. PERMANOVA and PCO were conducted using Primer v.6, while the RDA and GAMs were performed using the “Vegan” package [51] and the “MGCV” package [52] for R v.3.6.2 [53].

## Results

### General assemblage characterization

The average transect depth was 9.4 m (± 3.3), with the shallowest transect at Poca Esperanza (KNP, 3.5 m) and the deepest at Puerto Back (IE, 18 m). Horizontal visibility ranged from 6 m in the inland fjords to 20 m in sites exposed to the open ocean. Seawater temperature averaged 9.8 ^o^C (± 1.0), with the lowest temperature observed at Puerto Cook (IE, 8.14 ^o^C) and the highest at Isla Gaeta (KNP, 12.65 ^o^C). Salinity averaged 30.0 ppt (± 4.4), with the highest salinity observed at Isla Hornos (CH, 33.51 ppt) and the lowest at Poca Esperanza (KNP, 16.73 ppt). Rocky substrate was the dominant habitat type, covering 43.8% of the area surveyed, followed by rock with sand (14.9%), and rock with silt (9.9%). Among all sites, 46.3% were classified as sheltered, followed by wave-exposed stations (27.3%). The density of *M*. *pyrifera* ($\bar{X}$ = 4.8 ± 2.8) was significantly higher than the density of *Lessonia* spp. ($\bar{X}$ = 0.9 ± 1.3) (Wilcoxon rank sum test, W = 272.5, p < 0.001), which was absent in 26% of transects, while *M*. *pyrifera* was present in all transects.

We recorded 25 species of fishes, represented by 2,124 individuals from 13 families and 6 orders across the survey area (S1 Table). The expected number of fish species estimated by Chao 2 was 30.2 compared to the observed number, which was 25 (S1 Fig). The cumulative Chao 2 estimator curve reached an asymptote at 88 transects, suggesting that an adequate sampling effort was made. There were seven species > 20 cm TL mean observed on transects, which included pink cuskeel *Genypterus blacodes* (n = 1, 38 cm TL), the labrisomid blenny *Calliclinus geniguttatus* (n = 3, 26.5 ± 4.9 cm TL), two eel cods *Muraenolepis marmorata* and *M*. *orangiensis* (both n = 1, 25 cm TL), the South American eelpout *Austrolycus depressiceps* (n = 3, 24.7 ± 8.5 cm TL), the Patagonian redfish *Sebastes oculatus* (n = 1, 24 cm TL) and the southern hagfish *Myxine australis* (n = 4, 21.7 ± 7.6 cm TL) (S1 Table).

Nototheniidae was the most specious family with 8 species, accounting for 32% of all species observed, followed by Zoarcidae with 5 species, which accounted for an additional 20% of all species. Of the 25 species observed on transects, 64% are endemic to the Magellanic Province and an additional 12% are Magellanic, Subantarctic Islands endemics. Invertebrate feeders accounted for 52% of all fishes observed, while the remainder fed on fishes and invertebrates (S1 Table).

Nototheniidae accounted for 96% of total fish abundance and 94% of total fish biomass on transects (Table 1). *Patagonotothen tessellata* was the most abundant species, accounting for 29% of total abundance and 35% of total biomass, followed by *P*. *squamiceps*, with 23% of total abundance and 14% of total biomass. *P*. *cornucola* was the most frequently encountered species, occurring in 70% of all transects and contributing to 18% of total abundance and 28% of total biomass.

**Table 1:** Summary statistics for species observed on quantitative transects.

Family/Species	Num m^-2^ (sd)	% num	g m^-2^ (sd)	% biomass	% freq
**Nototheniidae**					
*Patagonotothen tessellata*	0.102 (0.28)	29.18	1.82 (5.39)	35.31	35
*Patagonotothen squamiceps*	0.082 (0.14)	23.46	0.72 (1.12)	13.97	56
*Patagonotothen cornucola*	0.064 (0.08)	18.31	1.45 (1.94)	28.13	70
*Patagonotothen sima*	0.061 (0.18)	17.45	0.14 (0.30)	2.72	41
*Paranotothenia magellanica*	0.016 (0.04)	4.58	0.26 (0.76)	5.04	22
*Patagonotothen brevicauda*	0.005 (0.03)	1.43	0.11 (0.43)	2.13	13
*Patagonotothen* sp.	0.003 (0.01)	0.86	0.02 (0.08)	0.39	7
*Patagonotothen longipes*	0.002 (0.01)	0.57	0.09 (0.41)	1.75	7
*Cottoperca trigloides*	0.002 (0.01)	0.57	0.26 (1.37)	5.04	8
**Agonidae**					
*Agonopsis chiloensis*	0.002 (0.01)	0.57	0.01 (0.06)	0.19	7
**Syngnathidae**					
*Leptonotus blainvilleanus*	0.001 (<0.01)	0.29	0.01 (0.04)	0.19	5
**Harpagiferidae**					
*Harpagifer bispinis*	0.001 (0.01)	0.29	0.01 (0.02)	0.19	5
**Zoarcidae**					
*Austrolicus depressiceps*	<0.001 (<0.01)	0.29	0.02 (0.13)	0.39	3
*Crossostomus chilensis*	0.001 (<0.01)	0.29	0.015 (0.109)	0.29	3
*Pogonolycus marinae*	<0.001 (<0.01)	0.17	0.002 (0.015)	0.04	2
*Piedrabuenia ringueleti*	<0.001	0.17	0.002 (0.025)	0.04	1
*Dadyanos insignis*	<0.001	0.17	0.003 (0.029)	0.06	1
**Myxinidae**					
*Myxine australis*	0.001 (<0.01)	0.29	0.01 (0.07)	0.19	2
**Labrisomidae**					
*Calliclinus geniguttatus*	<0.001 (<0.01)	0.17	0.10 (0.75)	1.94	2
**Tripterygiidae**					
*Helcogrammoides cunninghami*	<0.001 (<0.01)	0.17	0.003 (0.025)	0.06	2
**Ophidiidae**					
*Genypterus blacodes*	<0.001	0.17	0.036 (0.398)	0.70	1
**Sebastidae**					
*Sebastes oculatus*	<0.001	0.17	0.032 (0.353)	0.62	1
**Muraenolepididae**					
*Muraenolepis marmorata*	<0.001	0.17	0.015 (0.162)	0.29	1
*Muraenolepis orangiensis*	<0.001	0.17	0.015 (0.162)	0.29	1
**Liparidae**					
*Careproctus pallidus*	<0.001	0.17	0.001 (0.012)	0.02	1

Values are mean number of individuals m^-2^ and biomass (g m^-2^) with standard deviations in parentheses; % frequency of occurrence (freq).

**Fig 2:**
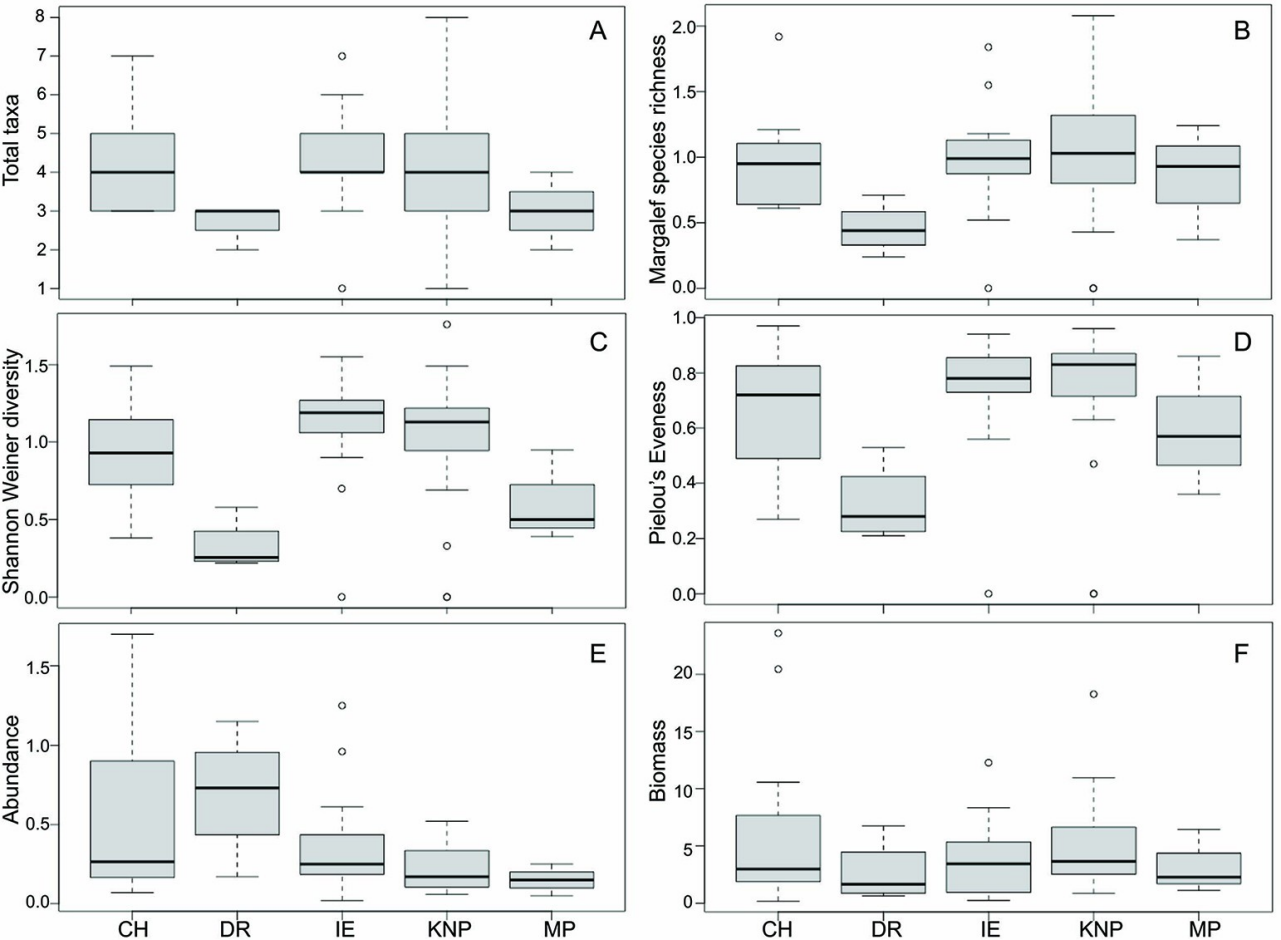
Fish assemblage characteristics among sampling stations. A. Total taxa, B. Species richness (Margalef), C. Shannon-Weiner Diversity, D. Pielou’s Evenness, E. Number of individuals m^-2^, F. Biomass (g m^-2^).

**Fig 3:**
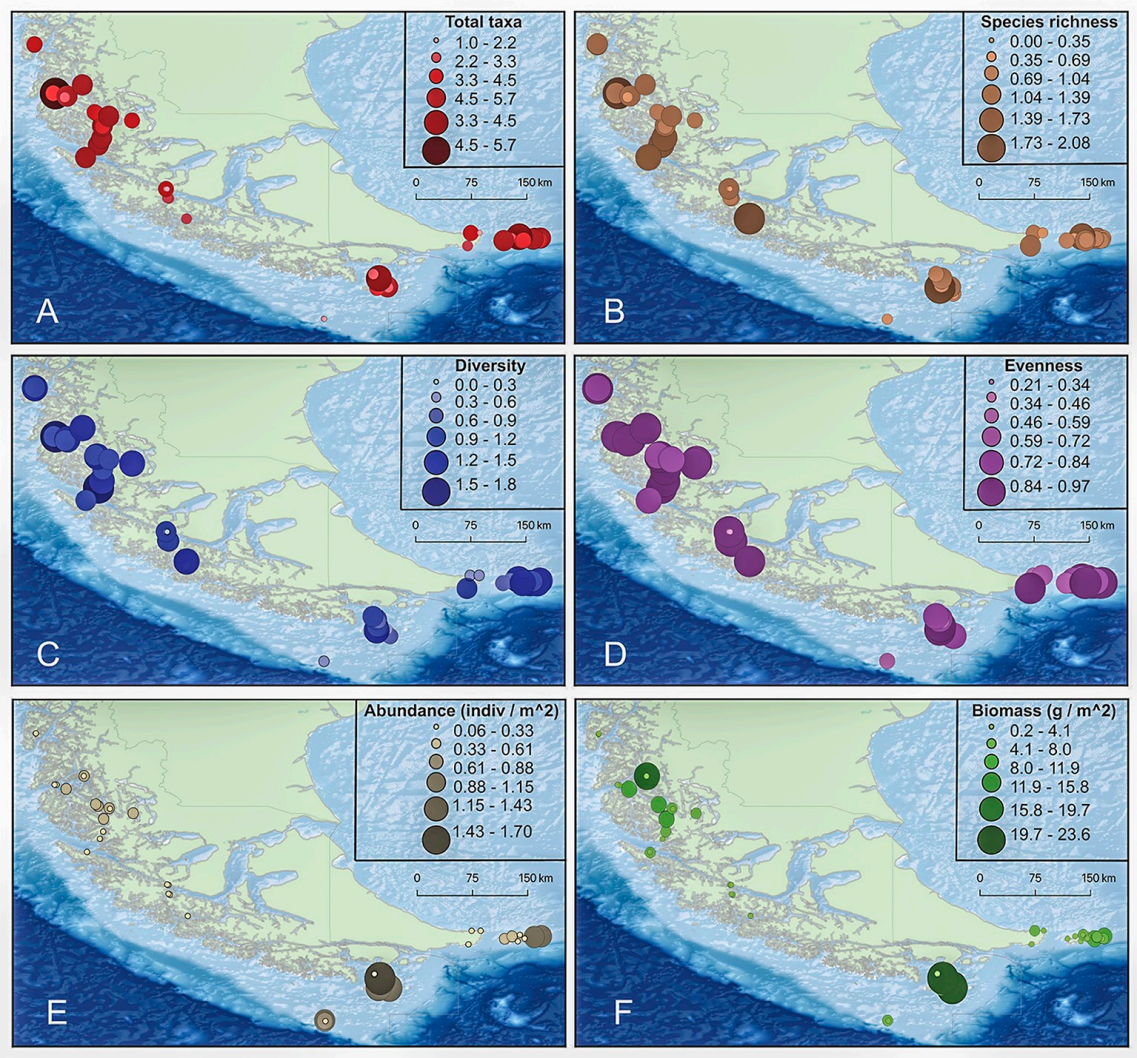
A. Total taxa, B. Margalef species richness, C. Shannon-Weiner Diversity, D. Pielou’s Evenness, E. Abundance, F. Biomass between locations. Boxes represent 25th, median and 75th percentiles, and upper and lower quartiles. Basemap derived from GEBCO Compilation Group (2020) GEBCO 2020 Grid (doi:10.5285/a29c5465-b138-234de053-6c86abc040b9). Processing and assembly of the Global Self-consistent, Hierarchical, High-resolution Geography Database for shoreline data from [35].

The average number of taxa per station 3.97 (± 1.35) was not significantly different among sampling locations although the results are suggestive (χ^2^ = 9.09, p = 0.06) (Figs 2A and 3A). Total average Margalef species richness was 0.96 (± 0.44) and did also not different among locations (χ^2^ = 7.66, p = 0.11) (Figs 2B and 3B). Diversity was significantly different among locations (χ^2^ = 15.00, p = 0.01), with the highest average diversity at IE ($\bar{X}$ = 1.09 ± 0.36) and the lowest at DR ($\bar{X}$ = 0.33 ± 0.17) (Figs 2C and 3C). Evenness was also significantly different among locations (χ^2^ = 9.84, p = 0.04), with the highest evenness at KNP and IE and the lowest at DR (Figs 2D and 3D). The total average number of individuals m^-2^ was 0.35 (± 0.36), which differed significantly among locations (χ^2^ = 9.43, p = 0.05), with the highest average abundance at DR ($\bar{X}$ = 0.70 ± 0.40) and the lowest at MP ($\bar{X}$ = 0.15 ± 0.10), followed by KNP ($\bar{X}$ = 0.22 ± 0.14) (Figs 2E and 3E). Average biomass was highest at CH ($\bar{X}$ = 6.35 ± 7.80) and lowest at DR ($\bar{X}$ = 2.67 ± 2.79). However, these differences were not significant (χ^2^ = 2.61, p = 0.63) (Figs 2F and 3F).

### Spatial patterns of fish assemblages and diversity

**Fig 4:**
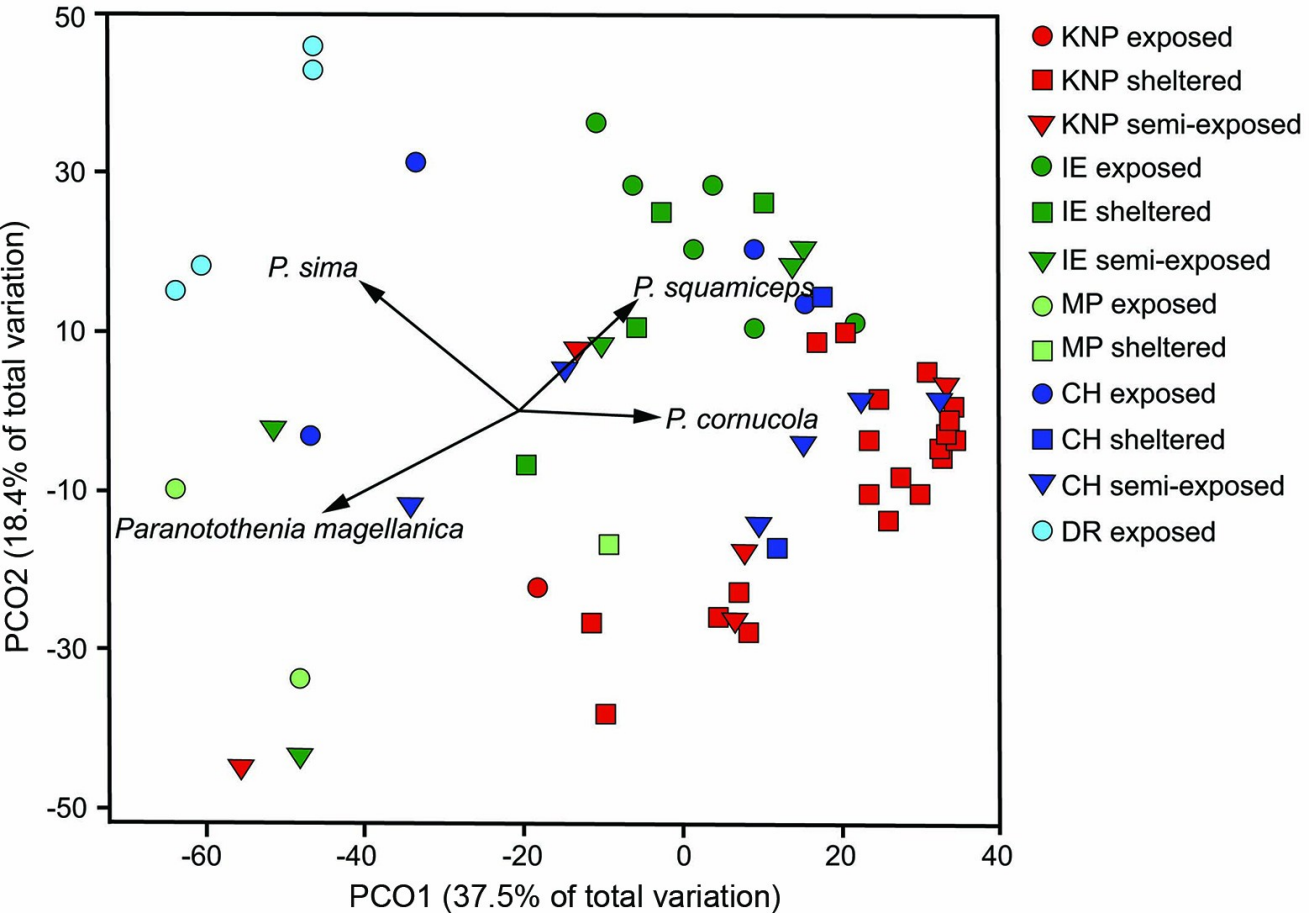
Principal coordinates analysis of fish assemblages based on numerical abundance by location and exposure. Data were ln(x+1)-transformed prior to analyses. Vectors are the primary taxa driving the ordination (Pearson correlations ≥ 0.5). *P*. = *Patagonotothen* spp.

There was a significant difference in fish assemblage structure among locations and exposures; however, the interaction of location and exposure was not significant (Table 2). Fish assemblage structure at exposed stations was significantly different between DR and CH (t = 2.32, p = 0.025) and between DR and IE (t = 4.16, p = 0.009) but not between CH and IE (t = 1.40, p = 0.117). Fish assemblage structure at sheltered stations was significantly different between KNP and IE (t = 1.82, p = 0.008) and marginally different between KNP and MP (t = 1.78, p = 0.05). Stations were well separated in ordination space, with PCO1 accounting for 37.5% of the total variation in fish assemblage structure among locations and exposures, while PCO2 explained an additional 18.4% of the variation (Fig 4). Exposed stations from IE and CH clustered together and were highly concordant with one another. Sheltered stations were clustered along the higher end of PCO1, with sheltered KNP stations highly concordant. *Patagonotothen cornucola* was most closely correlated with sheltered and semi-exposed stations of KNP and CH, while *P*. *sima* was most closely correlated with exposed stations of DR and CH. *Paranotothenia magellanica* was most closely correlated with exposed stations, primarily with MP and semi-exposed stations at CH and IE, while *P*. *squamiceps* was most closely correlated with stations of IE with different exposure.

**Table 2:** Comparison of fish assemblage composition based on density (number of individuals m^-2^) between locations and exposures with permutation-based multivariate analysis of variance (PERMANOVA).

Source	df	MS	Pseudo-F	P(perm)	
Location	4	5335.8	3.465	0.002	
Exposure	2	3591.3	2.332	0.014	
Location x exposure	5	2028.9	1.318	0.137	
Residuals	49	1539.9			
Total	60				
Pair-wise comparison					
Exposed Locations	t	P(perm)	Sheltered Locations	t	P(perm)
KNP, CH	1.179	0.191	KNP, CH	1.071	0.318
KNP, DR	2.916	0.216	**KNP, IE**	**1.821**	**0.008**
KNP, IE	2.187	0.140	**KNP, MP**	**1.783**	**0.050**
KNP, MP	2.317	0.345	CH, IE	1.091	0.392
**CH, DR**	**2.317**	**0.025**	CH, MP	1.340	0.658
CH, IE	1.402	0.117	IE, MP	1.965	0.213
CH, MP	2.171	0.058	Semi-exposed Locations		
**DR, IE**	**4.160**	**0.009**	KNP, CH	0.691	0.770
DR, MP	3.175	0.066	KNP, IE	0.787	0.584
IE, MP	3.718	0.036	CH, IE	1.002	0.394

Significant pairwise comparisons (*P* ≤ 0.05) are indicated in bold.

**Fig 5:**
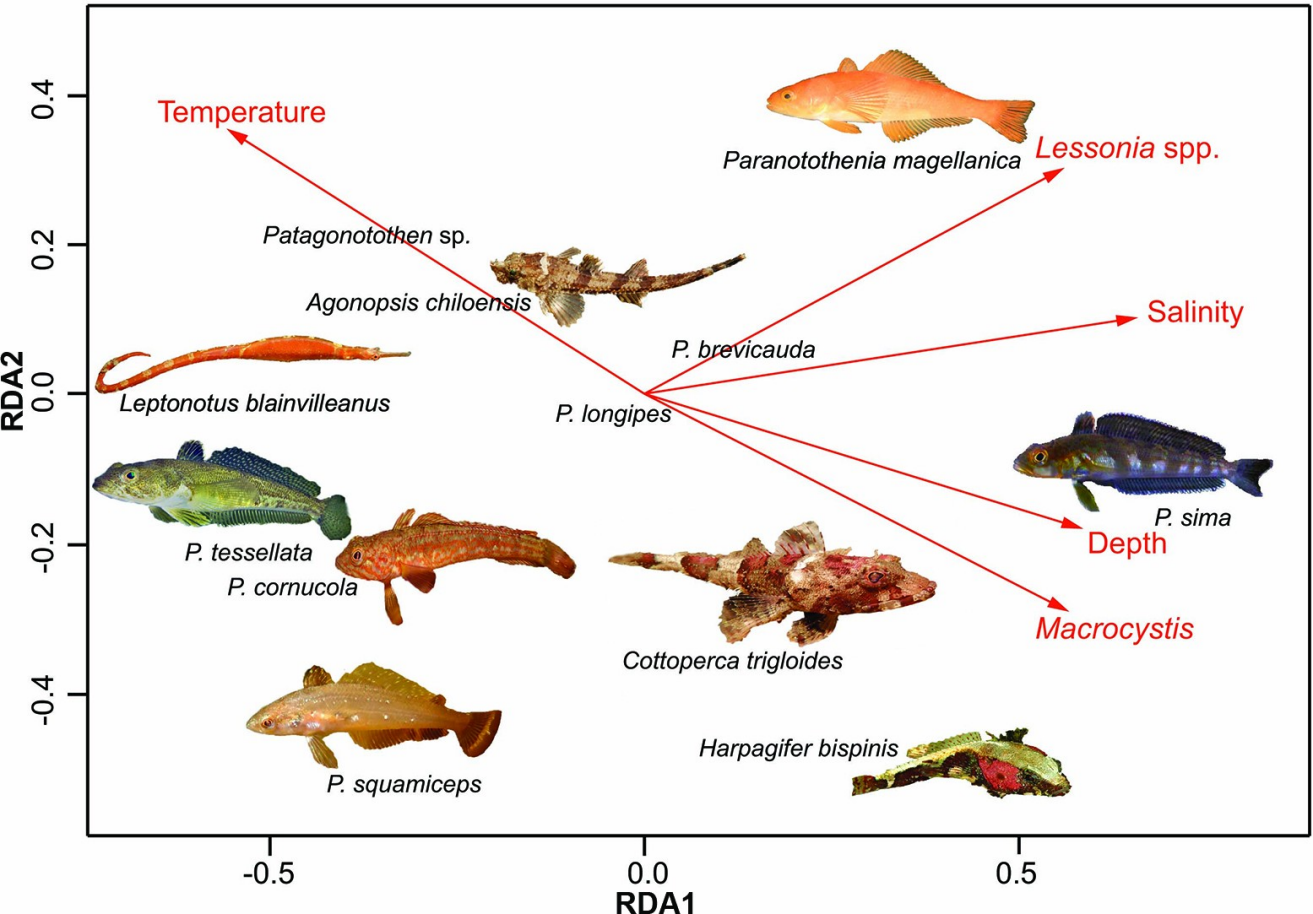
Redundancy Analysis (RDA) ordination biplot with environmental factors influencing fish assemblage structure. Species with low abundance were not included in this biplot. *P*. = *Patagonotothen* spp.

The explanatory variables accounted for 19% of total model variation, with RDA1 explaining 37% of the fish assemblage and environmental relationship and RDA2 explaining an additional 24% (Table 3). On the lower left side of the biplot, *P*. *tessellata*, *P*. *cornucola* and especially *P*. *squamiceps* were associated with low density or an absence of *Lessonia* spp. and low salinity, while *P*. *magellanica* was more strongly associated with higher densities of *Lessonia* spp. *P*. *sima* showed a stronger relationship with depth, high salinity, and *M*. *pyrifera* density. *Harpagifer bispinis* and *Cottoperca trigloides* were associated with high densities of *M*. *pyrifera* (Fig 5). Abiotic factors (salinity, temperature, and depth) accounted for 11% of the variance in fish assemblage, with biotic factors (densities of *M*. *pyrifera* and *Lessonia* spp.) accounting for an additional 8% (Table 3). A large percentage of variance was not explained by any of the predictors analyzed (residual = 81%).

**Table 3:** Results of Redundancy Analysis (RDA). A. RDA on sqrt-transformed data on fish abundance with environmental variables (salinity (ppt), temperature (^o^C), depth (m), *Macrocystis pyrifera* stipe density, and *Lessonia* spp. stipe density). B. RDA with variance partition performed to quantify the contribution of abiotic and biotic factors.

A. Axes	Axis 1	Axis 2	Axis 3	
Eigenvalues	1.53	0.99	0.65	
Proportion explained	0.37	0.24	0.16	
Cumulative proportion	0.37	0.61	0.77	
B. Partition	R^2^adj	% Explained	F	P
Abiotic (Salinity (ppt) + Temperature (^o^C) + Depth (m))	0.11	11.0	2.2	0.001
Biotic (*Lessonia* spp. density + *Macrocystis* density)	0.08	8.0	2.6	0.001

**Fig 6:**
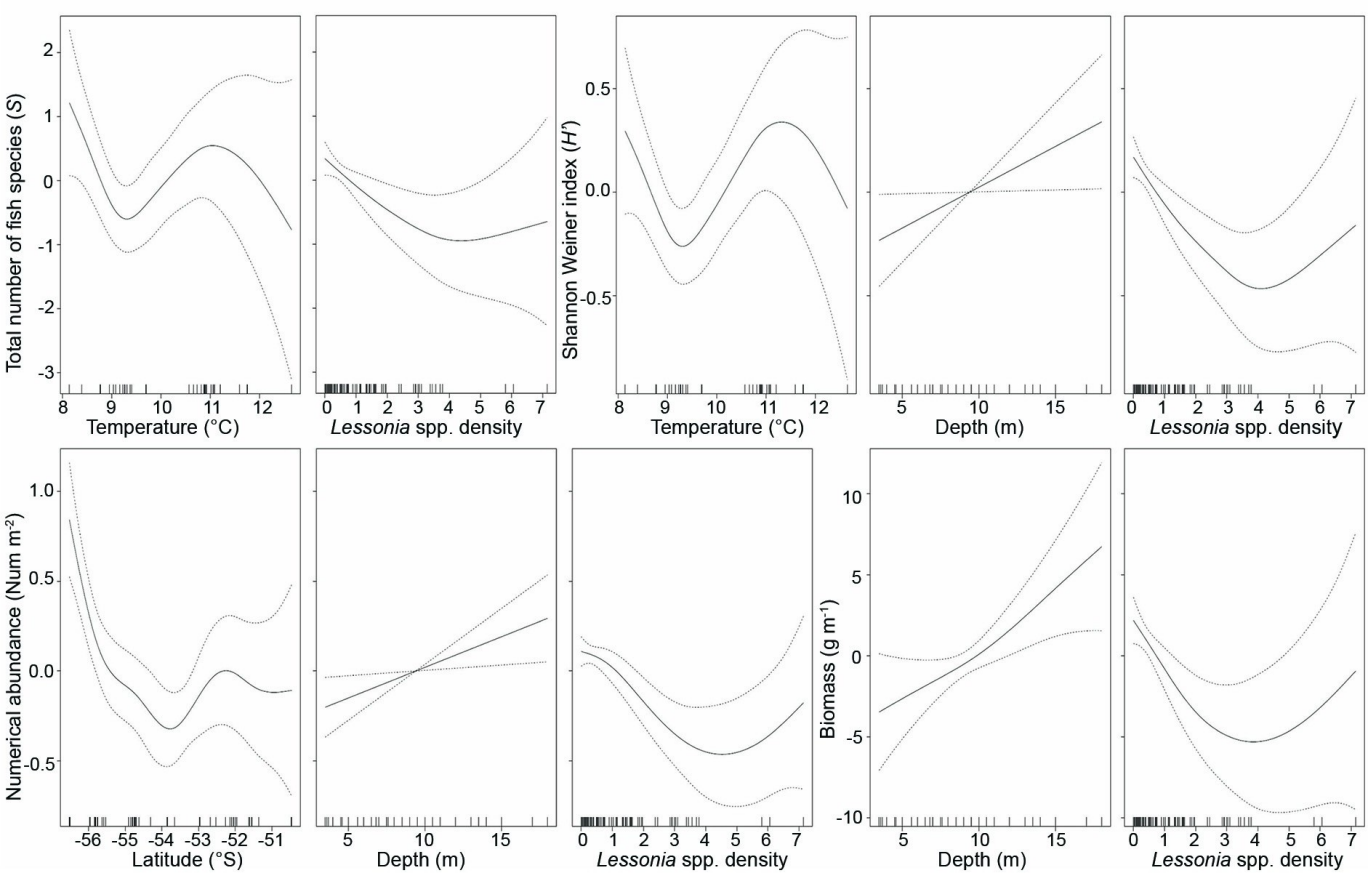
Smoothed estimates (solid line) for the environmental predictors temperature, *Lessonia* spp. density, depth and latitude as obtained by the best-fitting Generalized Additive Models (GAMs) for the total number of fish species (*S*), Shannon-Weiner index (*H*’), numerical abundance and biomass. Dashed lines are 95% confidence intervals. Tick marks on the x-axis are sampled data points.

All environmental variables were included in our GAM models since their VIF values were < 10 (S2 Table). The best-fitting GAM model for species richness (*S*) included exposure, bottom type, interaction of latitude and longitude, temperature, depth, and *Lessonia* spp. density (S3 Table). These predictors explained 41.9% of the variation in *S*, with temperature and *Lessonia* spp. density being significant factors in the model. Diversity (*H*’) was significantly influenced by the interaction of latitude and longitude, temperature, depth, and *Lessonia* spp. density. These variables along with exposure and bottom type explained 44% of the variation in *H*’. Abundance and biomass were significantly influenced by depth and *Lessonia* spp. density, with latitude also having a significant influence on abundance. These variables along with exposure and bottom type explained 57.5% and 37.6% of the variation in abundance and biomass, respectively (S3 Table). Temperature response curves showed the highest *S* and *H*’ at 8 ^o^C and 11 ^o^C, respectively (Fig 6). The *Lessonia* spp. density response curves showed *S*, *H*’, abundance, and biomass decreasing with *Lessonia* spp. density between 0–4 stipes m^-2^. The increase of *S*, *H*’, abundance, and biomass with *Lessonia* spp. density between 5–7 stipes m^-2^ should be interpreted as preliminary because of our small sample size at these densities. The depth response curves showed that *H*’, abundance, and biomass increased with increasing depth. The latitude response curve showed that abundance decreased with latitude between 56^o^S– 54^o^S and increased slightly between 53^o^S– 52^o^S (Fig 6).

## Discussion

The kelp forests of Southern Patagonia have some of the lowest anthropogenic disturbances on earth. Much of the coastal area is dominated by this important marine habitat, which plays a key role in structuring the nearshore communities of the region [6, 25, 32, 33]. Here we present the first spatially extensive study of kelp forest-associated fishes of the southern cone of South America conducted by quantitative underwater transects. The number of species recorded (25) is higher than previous studies for the region, which ranged from 6 to 19 taxa [6, 28–33]. However, species richness depends on the sampling method, and with the exception of Friedlander et al. [6, 32, 33] all previous studies were conducted with extractive methods (e.g., gillnets, holdfast collections). Taking into consideration the sampling bias, the overall number of fish species from previous studies (32 species) is concordant with the expected number of species estimated in our non-invasive visual transects. Most of the fishes previously described as kelp-forest species were recorded on our transects with the exception of the rockcod (*Eleginops maclovinus*) and the pike icefish (*Champsocephalus esox*). A plausible explanation for the absence of these species on visual transects could be related to the use in previous studies of trammel nets deployed overnight, since *E*. *maclovinus* inhabits bottom areas with silt and cobbles close to *M*. *pyrifera* forests [38] and *C*. *esox* has nocturnal habits [28]. However, a larger sampling effort could reveal species that were observed using other methods. The total number of species recorded in this study was similar to that reported by Pérez-Matus et al. [54], who observed ~ 26 species on transects of *M*. *pyrifera* and *Lessonia trabeculata* from the Pacific coast of northern Chile. Considering the gradual decrease in richness of littoral fishes along the Chilean coast towards higher latitudes [55], the high number of species recorded in kelp forests of Southern Patagonia is remarkable, and likely related to our extensive sampling effort, which was able to detect cryptic species from the families Syngnathidae (*Leptonotus blainvilleanus*), Liparidae (*Careproctus pallidus*), and Tripterygiidae (*Helcogrammoides cunninghami*). We also extend the known distribution of three species toward higher latitudes (*H*. *cunninghami*, *Calliclinus geniguttatus*, *Piedrabuenia ringueleti*) not previously recorded in this region. Percentage of species endemic to the Magellanic Province was high (60%) when compared to the total endemic species pool in the province (15%) [26]. According to Cousseau et al. [26], the fish families that best represent endemicity in the Magellanic Province are Nototheniidae and Zoarcidae, which were also the most specious families in our study. These families have some of the most rapid speciation rates among marine taxa, which highlights this region as a crucible of genetic biodiversity [56]. In our study, the notothenid genus *Patagonotothen* was the most species-rich genus and was also the most important in terms of abundance, biomass, and frequency of occurrence.

Five species of the genus *Patagonotothen* are restricted to shallow waters (≤ 40 m) and two of the most important species recorded in our study, *Patagonotothen sima* and *P*. *squamiceps*, have maximum depths ≤ 16 m [57], which correspond to the lower portion of the kelp zone. Early life stages of *Patagonotothen* species were found to be the dominant group associated with *M*. *pyrifera* kelp forests [58], highlighting its importance as a nursery area for these fishes. In addition, *Patagonotothen* species play a key role in the trophic ecology of shallow coastal waters of this region as they feed on benthic and zooplankton organisms [59–62], but they also are an important prey item for top-level predators such as seabirds and marine mammals [63–65]. Therefore, the *Patagonotothen* species represent an important link between lower (invertebrates) and higher (seabirds and marine mammals) levels of the food web.

Size ranges revealed an assemblage of small-sized fishes, with few specimens > 20 cm. The small-sized fish assemblage consisted mainly of *Patagonotothen* species [66]. While our sampling effort was limited by the difficult access to these remote areas, the general lack of knowledge on fish diversity and distribution within this region makes our results an important contribution in better understanding the biogeography of kelp forest fishes of Southern Patagonia and serves as a value baseline for future investigation.

Our results show differences in fish assemblage structure among locations and exposures, with the Diego Ramírez Islands (DR) significantly different from exposed stations of the Cape Horn Archipelago (CH) and Isla de los Estados (IE). DR had the lowest diversity, evenness, and biomass among all the locations we sampled, but the highest abundance, which was driven primarily by the high number of individuals of *Patagonotothen sima* that formed small schools among kelp fronds in areas exposed to strong wave action. Similarly, Moreno & Jara [28] noted that *P*. *sima* inhabited mid-water kelp fronds, except during their reproductive period in winter when they migrated to the holdfasts. Taxon richness was highest at Isla Lobos (8 species) in Kawésqar National Park (KNP) followed by Isla Wollaston at CH and Collnet Islet at IE, both with 7 species. We frequently observed *P*. *cornucola* in sheltered areas around KNP and at CH, where they were found in semi-exposed areas. *Paranotothenia magellanica* was associated with exposed and semi-exposed stations, where they were regularly observed in the canopy of *Lessonia* spp. This is supported by the redundancy analyses, showing *Lessonia* spp. density being a major factor in explaining *P*. *magellanica* abundance. On the other hand, Vanella et al [30] found that the removal of *M*. *pyrifera* forests resulted in a decrease in the abundance of *P*. *magellanica* in the Beagle Channel [30]. Our analysis showed that *P*. *squamiceps* was associated with areas of low density of *Lessonia* spp. We frequently observed *P*. *squamiceps* associated with stipes and fronds of *M*. *pyrifera*.

We found a significant negative correlation between *Lessonia* spp. density and the number of fish species (*S*), diversity (*H*’), abundance, and biomass. These negative relationships with fish assemblage metrics and *Lessonia* spp. are influenced by *P*. *tessellata*, *P*. *squamiceps* and *P*. *cornucola*, which are the most important species in terms of abundance and biomass in our study and were most closely associated with *M*. *pyrifera* forests. Friedlander et al. [32] recorded > 18,000 invertebrate individuals on a single *M*. *pyrifera* specimen at IE, especially amphipods that are often the main component of fish diets in the region [28, 61]. Therefore, *M*. *pyrifera* fronds likely provide more food and refuge for fishes compared to the smaller and less structurally complex *Lessonia* fronds [20].

Depth was positively related to *H*’, abundance, and biomass in our GAM models, with all of these metrics greater at deeper depths, while temperature significantly influenced *S* and *H*’. Previous studies in southern South America have indicated that depth is an important factor influencing diversity and community structure of invertebrates associated with *M*. *pyrifera* forests at Punta Santa Ana in the Strait of Magellan [67] and Kidney Island, Falkland/Malvinas Islands [68]. Depth is also important in fish diversity and abundance in high latitude kelp forests of the Northern Hemisphere [69]. Temperature has also been shown to influence the abundance of *P*. *magellanica* and *P*. *tessellata* in the Beagle Channel, which could affect the activity patterns of these species [30, 70]. In our study, the highest *H*’ occurred at IE in areas with the lowest temperature and deepest depths. We also found increases in *H*’ and *S* at 11°C in areas of KNP such as Isla Lobos, where the highest species richness was observed.

This study demonstrated that the fish assemblages associated with kelp forests in Southern Patagonia are influenced by a number of biotic and abiotic factors. Although the overall variance explained was low (only 11% and 8%, respectively), these results are consistent with other studies in the region. Our study highlights the high variability in these assemblages and points to several important environmental variables, as well as intraspecific interactions that might influence these assemblages [71].

Recent research has shown that kelp forests populations in Southern Patagonia that were subjected to high turbidity exhibit adaptation to photosynthesize by shade adapted characteristics, which may make it possible for them to acclimate to certain environmental impacts from climate change (e.g., warming, ice melting, and glacial retreat) [72]. In addition, the southern cone of South America is predicted to warm more slowly than other regions of the world and currently is not showing signs of tropicalization [73]. Therefore, Southern Patagonia may be less impacted by climate change compared with kelp forests elsewhere around the world. However, a combination of environmental changes has been detected in this region through regional climatic-oceanographic anomalous events (e.g., El Niño Southern Oscillation, Southern Annular Mode), hydrological changes (e.g., decreasing pattern of rainfall in watersheds and into fjords), and more frequent harmful algal blooms (HABs), which can eventually result in fish kills and major shifts in the food web structure [74, 75]. Anthropogenic activities, such as salmon farming could also modify the seasonal phytoplankton blooms and stimulate the growth of HAB in Southern Chile by overfertilization and feed additions in coastal salmon farms (e.g., ammonium input) [76]. Another concern is farmed salmon escapees, which are not native to Chile, and can impact native fishes through strong predatory pressure [77, 78]. For example, notothenioids were one of the main prey items of salmonids in Aysen Fjord [79]. Estimates suggest that more than 1 million salmonids escape annually from marine farms in Southern Chile, mainly due to weather conditions and technical and operational failures of net-pens [80]. In addition, antibiotic residues have been found in muscle samples from native fishes captured around salmon farms in Chiloe [81]. There are currently 58 aquaculture concessions approved and 176 new requests for concessions within the KNP area and this will likely only increase in the coming years [33]. In response to these impacts, the Argentine province of Tierra del Fuego recently banned salmon farming in open net pens, making Argentina the first country in the world to limit salmon farming.

In order to provide effective conservation of this unique ecosystem, it is essential to implement management actions that restrict the expansion of the salmon farming, establishment of marine protected areas and marine coastal areas of indigenous people, monitoring of spatio-temporal variability of environmental variables (e.g., chlorophyll-a, sea surface temperature, dissolved oxygen, salinity), and monitoring the main populations of kelp forest-associated fishes.

Our work paves the way for future research in seasonal patterns and other factors such as habitat structural complexity and fine-scale intraspecific variability in fish assemblages on these high latitude habitat-forming kelp forests. Since this is the first study from a vast area of the southern cone of South America, these results can provide important baseline information that can be used to compare future changes due to species distribution and abundance shifts, as well as improving the knowledge for the assessment and management of these species, particularly in the context of marine protected areas.

## Supporting information

S1 Fig Species accumulation curves using Sobs and expected number of species (Chao 2 estimator) for fish assemblages (error bars = SD). (TIF)

S1 Table Species observed during surveys in the Magellanic Province. Pisc = piscivore; Inv = invertivore. Size range in cm are from quantitative underwater transects. Family names in bold. *Magellanic endemic; ^+^ Magellanic, Subantarctic Is. endemic. Data on habitat are based on our personal observation and from previous work (Vanella et al. 2007; Fernández et al. 2012)
(DOCX)

S2 Table Explanatory variables used in this study. VIF: variance inflation factor; n/a: not assessed.
(DOCX)

S3 Table Best fitting generalized additive models for the total number of fish species, Shannon-Weiner diversity, Numerical abundance and biomass. edf: estimated degrees of freedom; AIC: Akaike information criterion; te: tensor product interaction; s: smooth term for predictor variables. *P* < 0.05 is indicated in bold.
(DOCX)

## References

[1] Clapperton CM, Sugden DE, Kaufman DS, McCulloch RD. The last glaciation in central magellan strait, southernmost chile. Quat Res. 1995;44: 133–148. doi: 10.1006/qres.1995.1058

[2] Hulton NRJ, Purves RS, McCulloch RD, Sugden DE, Bentley MJ. The last glacial maximum and deglaciation in southern South America. Quat Sci Rev. 2002;21: 233–241. doi: 10.1016/S0277-3791(01)00103-2

[3] Sudgen DE, Hulton N, Purves RS. 2002. Modelling the inception of the Patagonian icesheet. Quatern Int. 2002;95–96: 55–64. doi: 10.1016/S1040-6182(02)00027-7

[4] Silva N, Calvete C. Características oceanográficas físicas y químicas de canales australes chilenos entre el golfo de Penas y el estrecho de Magallanes (Crucero CIMAR Fiordo 2). Cienc Tecnol Mar. 2002;22: 23–88.

[5] Pantoja S, Iriarte JL, Daneri G. 2011. Oceanography of the Chilean Patagonia. Cont Shelf Res. 2011;31: 149–153. doi: 10.1016/j.csr.2010.10.013

[6] Friedlander AM, Ballesteros E, Bell TW, Giddens J, Henning B, Hüne M, et al. Marine biodiversity at the end of the world: Cape Horn and Diego Ramírez islands. PLoS One. 2018;13. doi: 10.1371/journal.pone.0189930 29364902 PMC5783361

[7] Antezana T. Hydrographic features of Magellan and Fuegian inland passages and adjacent subantarctic waters. Sci Mar. 1999;63: 23–34. doi: 10.3989/scimar.1999.63s123

[8] Miloslavich P, Klein E, Díaz JM, Hernández CE, Bigatti G, Campos L, et al. Marine biodiversity in the Atlantic and Pacific coasts of South America: Knowledge and gaps. PLoS One. 2011; 6. doi: 10.1371/journal.pone.0014631 21304960 PMC3031619

[9] Brown JH, Stevens GC, Kaufman DM. The geographic range: size, shape, boundaries, and internal structure. Annu Rev Ecol Syst. 1996;27: 597–623. doi: 10.1146/annurev.ecolsys.27.1.597

[10] Friedlander AM, Brown EK, Jokiel PL, Smith WR, Rodgers KS. Effects of habitat, wave exposure, and marine protected area status on coral reef fish assemblages in the Hawaiian archipelago. Coral Reefs. 2003;22: 291–305. doi: 10.1007/s00338-003-0317-2

[11] Luiz OJ, Mendes TC, Barneche DR, Ferreira CG, Noguchi R, Villaca RC, et al. Community structure of reef fishes on a remote oceanic island (St Peter and St Paul’s Archipelago, equatorial Atlantic): the relative influence of abiotic and biotic variables. Mar Freshwater Res. 2015;66: 739–749. doi: 10.1071/MF14150

[12] Zeng X, Tanaka KR, Mazur M, Wang K, Chen Y, Zhang S. Effects of habitat on reef fishes biodiversity and composition in rocky reefs. Aquat Biol. 2020;29: 137–148. doi: 10.3354/ab00731

[13] Barneche DR, Kulbicki M, Floeter SR, Friedlander AM, Allen AP. Energetic and ecological constraints on population density of reef fishes. Proc R Soc B. 2016;283: 20152186. doi: 10.1098/rspb.2015.2186 26791611 PMC4795013

[14] Quimbayo JP, Murilo SD, Kulbicki M, Mendes TC, Lamb RW, Johnson AF, et al. Determinants of reef fish assemblages in tropical Oceanic islands. Ecography. 2019;42: 77–87. doi: 10.1111/ecog.03506

[15] Levin PS, Hay ME. Responses of temperate reef fishes to alterations in algal structure and species composition. Mar Ecol Prog Ser. 1996;134: 37–47.

[16] Arkema KK, Reed DC, Schroeter SC. Direct and indirect effects of giant kelp determine benthic community structure and dynamics. Ecology. 2009;90: 3126–3137. doi: 10.1890/08-1213.1 19967868

[17] Steneck RS, Graham MH, Bourque BJ, Corbett D, Erlandson JM, Estes JA, et al. Kelp forest ecosystems: biodiversity, stability, resilience and future. Environ Conserv. 2002;29: 436–459. doi: 10.1017/S0376892902000322

[18] Johnson DW. Predation, habitat complexity, and variation in density-dependent mortality of temperate reef fishes. Ecology. 2006;87: 1179–1188. doi: 10.1890/0012-9658(2006)87[1179:phcavi]2.0.co;2 16761597

[19] Pérez-Matus A, Pledger S, Díaz FJ, Ferry LA, Vásquez JA. Plasticity in feeding selectivity and trophic structure of kelp forest associated fishes from northern Chile. Rev Chil Hist Nat. 2012;85: 29–48. doi: 10.4067/S0716-078X2012000100003

[20] Miller RJ, Page HM, Reed DC. Trophic versus structural effects of a marine foundation species, giant kelp (*Macrocystis pyrifera*). Oecologia. 2015;179: 1199–1209. doi: 10.1007/s00442-015-3441-0 26358195

[21] Holbrook SJ, Carr MH, Schmitt RJ, Coyer JA. Effect of giant kelp on local abundance of reef fishes: the importance of ontogenetic resource requirements. Bull Mar Sci. 1990;47: 104–114.

[22] Mora-Soto A, Palacios M, Macaya E, Gómez I, Huovinen P, Pérez-Matus A, et al. A High-Resolution Global Map of Giant Kelp (Macrocystis pyrifera) Forests and Intertidal Green Algae (Ulvophyceae) with Sentinel-2 Imagery. Remote Sens. 2020;12: 694. doi: 10.3390/rs12040694

[23] Buschmann AH, Vásquez JA, Osorio P, Reyes E, Filún L, Hernández-González MC, et al. The effect of water movement, temperature and salinity on abundance and reproductive patterns of *Macrocystis* spp. (Phaeophyta) at different latitudes in Chile. Mar Biol. 2004;145: 849–862. doi: 10.1007/s00227-004-1393-8

[24] Huovinen P, Leal P, Gómez I. Interacting effects of copper, nitrogen and UV radiation on the physiology of three south Pacific kelps. Mar Freshwater Res. 2010;61: 330–341. doi: 10.1071/MF09054

[25] Dayton PK. The structure and regulation of some South American kelp communities. Ecol Monogr. 1985;55: 447–468. doi: 10.2307/2937131

[26] Cousseau MB, Pequeño G, Mabragaña E, Lucifora LO, Martinéz P, Giussi A. 2019. The Magellanic Province and its fish fauna (South America): Several provinces or one? J Biogeogr. 2019;47: 220–234. doi: 10.1111/jbi.13735

[27] Hüne M. 2019. Lista sistemática actualizada de los peces de Chile. Versión 1.4. Checklist dataset. Global Biodiversity Information Facility. 2019. Available: 10.15468/er28jy

[28] Moreno CA, Jara HF. Ecological studies on fish fauna associated with *Macrocystis pyrifera* belts in the south of Fueguian Islands, Chile. Mar Ecol Prog Ser. 1984;15: 99–107.

[29] Ríos C, Arntz WE, Gerdes D, Mutschke E, Montiel A. Spatial and temporal variability of the benthic assemblages associated to the holdfasts of the kelp *Macrocystis pyrifera* in the Straits of Magellan, Chile. Polar Biol. 2007;31: 89–100. doi: 10.1007/s00300-007-0337-4

[30] Vanella F, Fernández D, Romero M, Calvo J. Changes in the fish fauna associated with a sub-Antarctic *Macrocystis pyrifera* kelp forest in response to canopy removal. Polar Biol. 2007;30: 449–457. doi: 10.1007/s00300-006-0202-x

[31] Cruz-Jiménez AM. Ensambles de peces en los bosques de kelp de *Macrocystis pyrifera* en el Canal Beagle, Tierra del Fuego: estructura comunitaria y variación espacio-temporal. PhD Thesis, Universidad Nacional de La Plata. 2019. Available from: http:sedici.unlp.edu.ar/handle/10915/79453

[32] Friedlander AM, Ballesteros E, Bell TW, Caselle JE, Campagna C, Goodell W, et al. Kelp forests at the uttermost part of the earth: 45 years later. PLoS One. 2020;15. doi: 10.1371/journal.pone.0229259 32160219 PMC7065750

[33] Friedlander AM, Ballesteros E, Goodell W, Hüne M, Muñoz A, Salinas de León P, et al. Marine communities of the newly created Kawésqar National Reserve, Chile: from glaciers to the Pacific Ocean. PLoS One. 2021;16. doi: 10.1371/journal.pone.0249413 33852615 PMC8046254

[34] Fernández DA, Ceballos S, Malanga G, Boy C, Vanella F. Buoyancy of sub Antarctic notothenioids including the sister linage of all other notothenioids (Bovichtidae). Polar Biol. 2012;35(1):99–106. doi: 10.1007/s00300-011-1037-7

[35] Wessel P, Smith WHF. A global, self-consistent, hierarchical, high-resolution shoreline database. J Geophys Res B Solid Earth. 1996;101: 8741–8743. doi: 10.1029/96jb00104

[36] Pais MP, Cabral HH. Effect of underwater visual survey methodology on bias and precision of fish counts: a simulation approach. Peer J. 6:e5378. doi: 10.7717/peerj.5378 30083471 PMC6071614

[37] Lloris D, Rucabado J. Ictiofauna del Canal Beagle (Tierra de Fuego), aspectos ecológicos y análisis biogeográfico (No. 8). Madrid; 1991.

[38] Reyes P, Hüne M. Peces del sur de Chile. Santiago: Ocho Libros; 2012.

[39] Froese R, Pauly D. FishBase. World Wide Web electronic publication. version 12/2019. 2019. Available: http://www.fishbase.org

[40] Fernández DA, Bruno DO, Llompart FM. Length-weight relationship of six notothenioid species from sub-Antarctic waters (Beagle Channel, Argentina). 2019;35: 597–599. doi: 10.1111/jai.13833

[41] Riccialdelli L, Bruno D. Medidas de parámetros ambientales y productividad primaria del Área Marina protegida Namuncura–banco Burdwood y aguas adyacentes. In: Dellabianca N., editor. Informe de Campaña “Namuncura–banco Burdwood”; 2015. pp. 5–8.

[42] Veccia M, Molinari G. Oceanografía física. In: Schejter L, Lovrich G., editors. Informe de Campaña: Banco Burdwood Buque Oceanográfico ARA ¨Puerto Deseado¨; 2017. pp. 162–198.

[43] Chao A, Colwell RK, Lin C.-W, Gotelli NJ. Sufficient sampling for asymptotic minimum species richness estimators. Ecology. 2009;90: 1125–1133. doi: 10.1890/07-2147.1 19449706

[44] Colwell RK. EstimateS version 9.1.0. 2016. Available: http://purl.oclc.org/estimates.

[45] Clarke KR, Gorley RN, Somerfield PJ, Warwick RM. Change in marine communities: an approach to statistical analysis and interpretation, 3rd edn. Plymouth, UK: Primer-e; 2014.

[46] Anderson MJ, Gorley RN, Clarke KR. PERMANOVA+ for PRIMER: Guide to Software and Statistical Methods. Plymouth, UK: Primer-e; 2008.

[47] Zamora E, Santana A. Características climáticas de la costa occidental de la Patagonia, entre las latitudes 46° 40’ y 56° 30’ S. An Inst Pat. 1979;10:109–154.

[48] Andrade S. Geomorfología costera y antecedentes oceanográficos físicos de la región de Magallanes, Chile (48° – 56° S). An Inst Pat. 1991;20:135–151.

[49] Borcard D, Gillet F, Legendre P. Numerical ecology with R: exploratory data analysis. New York, NY: Springer; 2011.

[50] Wood SN. 2017. Generalized Additive Models: An introduction with R, 2 edition. New York, NY: Chapman and Hall/CRC; 2017.

[51] Oksanen J, Blanchet FG, Friendly M, Kindt R, Legendre P, McGlinn G, et al. Vegan: Community ecology package. R package version 2.5–7. 2020. Available: https://cran.r-project.org/web/packages/vegan/vegan.pdf

[52] Wood SN. MGCV: Mixed GAM Computation Vehicle with automatic smoothness estimation. R package version 1.8–33. 2020. Available: https://cran.r-project.org/web/packages/mgcv/mgcv.pdf

[53] R Core Team. R: A language and environment for statistical computing. R Foundation for Statistical Computing, Vienna, Austria; 2020.

[54] Pérez-Matus A, Ferry-Graham L, Cea A, Vásquez J. Community structure of temperate reef fishes in kelp-dominated subtidal habitats of northern Chile. Mar. Freshwater Res. 2007;58: 1069–1085. doi: 10.1071/MF06200

[55] Ojeda FP, Labra FA, Muñoz AA. Biogeographic patterns of Chilean littoral fishes. Rev Chil Hist Nat. 2000;73: 625–641. doi: 10.4067/S0716-078X2000000400007

[56] Rabosky DL, Chang J, Title PO, Cowman PF, Sallan L, Friedman M, et al. An inverse latitudinal gradient in speciation rate for marine fishes. Nature. 2018:559: 392–395. doi: 10.1038/s41586-018-0273-1 29973726

[57] Eastman JT. Bathymetric distribution of notothenioid fishes. Polar Biol. 2017;40:2077–2095. doi: 10.1007/s00300-017-2128-x

[58] Bruno DO, Victorio MF, Acha EM, Fernández DA. Fish early life stages associated with giant kelp forests in sub-Antarctic coastal waters (Beagle Channel, Argentina). Polar Biol. 2018;41:365–375. doi: 10.1007/s00300-017-2196-y

[59] Salas-Berrios F, Valdés-Aguilera J, Landaeta MF, Bustos CA, Pérez-Vargas A, Balbontín F. Feeding habits and diet overlap of marine fish larvae from the peri-Antarctic Magellan region. Polar Biol. 2013;36: 1401–1414. doi: 10.1007/s00300-013-1359-8

[60] Hüne M, Vega R. Spatial variation in the diet of *Patagonotothen tessellata* (Pisces, Nototheniidae) from the fjords and channels of southern Chilean Patagonia. Polar Biol. 2015;38: 1613–1622. doi: 10.1007/s00300-015-1726-8

[61] Hüne M, Vega R. Feeding habits in two sympatric species of Notothenioidei, *Patagonotothen cornucola* and *Harpagifer bispinis*, in the Chilean Patagonian channels and fjords. Polar Biol. 2016;39: 2253–2262. doi: 10.1007/s00300-016-1892-3

[62] Hüne M, Davis E, Murcia S, Gutiérrez D, Haro D. 2018. Trophic relationships of a subtidal fish assemblage in the Francisco Coloane Coastal Marine Protected Area, southern Chilean Patagonia. Polar Res. 2018;37. doi: 10.1080/17518369.2018.1435107

[63] Riccialdelli L, Newsome SD, Dellabianca NA, Bastida R, Fogel ML, Goodall RNP. Ontogenetic diet shift in Commerson’s dolphin (*Cephalorhnychus commersonii commersonii*) off Tierra del Fuego. Polar Biol. 2013;36: 617–627. doi: 10.1007/s00300-013-1289-5

[64] McInnes JC, Jarman SN, Lea M.-A, Raymond B, Deagle BE, Phillips RA, et al. DNA metabarcoding as a marine conservation and management tool: A circumpolar examination of fishery discards in the diet of theatened albatrosses. Front Mar Sci. 2017;4: 277. doi: 10.3389/fmars.2017.00277

[65] Haro D, Sabat P, Arreguín-Sánchez F, Neira S, Hernández-Padilla J. Trophic role of the humpback whale (*Megaptera novaeangliae*) in the feeding area of Magellan Strait, Chile. Ecol Indic. 2020;109. doi: 10.1016/j.ecolind.2019.105796

[66] Eastman JT. An analysis of maximum body size and designation of size categories for notothenioid fishes. Polar Biol. 2019;42:1131–1145. doi: 10.1007/s00300-019-02502-7

[67] Cárdenas CA, Montiel A. The influence of depth and substrate inclination on sessile assemblages in subantarctic rocky reefs (Magellan region). Polar Biol. 2015;38: 1631–1644. doi: 10.1007/s00300-015-1729-5

[68] Beaton EC, Küpper FC, van West P, Brewin P, Brickle P. The influence of depth and season on the benthic communities of a *Macrocystis pyrifera* forest in the Falkland Islands. Polar Biol. 2020;43: 573–586. doi: 10.1007/s00300-020-02662-x

[69] Efird T, Konar B. Habitat characteristics can influence fish assemblages in high latitude kelp forests. Environ Biol Fish. 2014;97: 1253–1263. doi: 10.1007/s10641-013-0211-x

[70] Vanella FA, Calvo J. Influence of temperature, habitat and body mass on routine metabolic rates of Subantarctic teleosts. Sci Mar. 2005;69: 317–323. doi: 10.3989/scimar.2005.69s2317

[71] Moller AP, Jennions MD. How much variance can be explained by ecologists and evolutionary biologists. Oecologia. 2002;132: 492–500. doi: 10.1007/s00442-002-0952-2 28547634

[72] Palacios M, Osman D, Ramírez J, Huovinen P, Gómez I. Photobiology of the giant kelp *Macrocystis pyrifera* in the land-terminating glacier fjord Yendegaia (Tierra del Fuego): A look into the future? Sci Total Environ. 2021;751: 141810. doi: 10.1016/j.scitotenv.2020.141810 32882566

[73] Vergés A, Steinberg PD, Hay ME, Poore AGB, Campbell AH, Ballesteros E, et al. The tropicalization of temperate marine ecosystems: Climate-mediated changes in herbivory and community phase shifts. Proc R Soc B Biol Sci. 2014;281: 20140846. doi: 10.1098/rspb.2014.0846 25009065 PMC4100510

[74] Iriarte JL, González HE, Nahuelhual L. Patagonian fjord ecosystems in southern Chile as a highly vulnerable region: problems and needs. AMBIO. 2010;39: 463–466. doi: 10.1007/s13280-010-0049-9 21090000 PMC3357667

[75] Iriarte JL. 2018. Natural and human influences on marine processes in Patagonian subantarctic coastal waters. Front Mar Sci. 2018;5: 360. doi: 10.3389/fmars.2018.00360

[76] Iriarte JL, Quiñones RA, González RR. 2005. Relationship between biomass and enzymatic activity of a bloom-forming dinoflagellate (Dinophyceae) in southern Chile (41S): A field approach. J Plankton Res. 2005;27: 159–166. doi: 10.1093/plankt/fdh167

[77] Soto D, Jara F, Moreno CA. Escaped salmon in the inner seas, southern Chile: facing ecological and social conflicts. Ecol Appl. 2001;11: 1750–1762. doi: 10.1890/1051-0761(2001)011[1750:ESITIS]2.0.CO;2

[78] Thomas F, Espíndola M, Vega A, Cabezas L, Hüne M, Avaria S, et al. Evaluación y análisis de la biodiversidad marina y continental afectada por las actividades de acuicultura (1era Etapa). FIP 2014–48. Valparaíso, Chile; 2017.

[79] Niklitschek EJ, Toledo P. 2011. Evaluación cuantitativa del estado trófico de salmónidos de vida libre en el Fiordo Aysén, XI Región. FIP 2008–30. Puerto Montt, Chile 2011. Available: https://www.subpesca.cl/fipa/613/articles-89240_informe_final.pdf

[80] Sepúlveda M, Arismendi I, Soto D, Jara F, Farías F. Escaped farmed salmon and trout in Chile: incidence, impacts, and the need for an ecosystem view. Aquac Environ Interact. 2013;4: 273–283. doi: 10.3354/aei00089

[81] Fortt A, Cabello F, Buschmann A. Residues of tetracycline and quinolones in wild fish living around a salmon aquaculture center in Chile. Rev Chilena de Infectol. 2007;24: 14–18. doi: 10.4067/s0716-10182007000100002 17369965

