# Supplementary figures and images for: Assemblage structure and spatial diversity patterns of kelp forest-associated fishes in Southern Patagonia
Source: PLoS One. 2021 Sep 20;16(9):e0257662. doi: 10.1371/journal.pone.0257662 (PMC8452001; doi:10.1371/journal.pone.0257662)

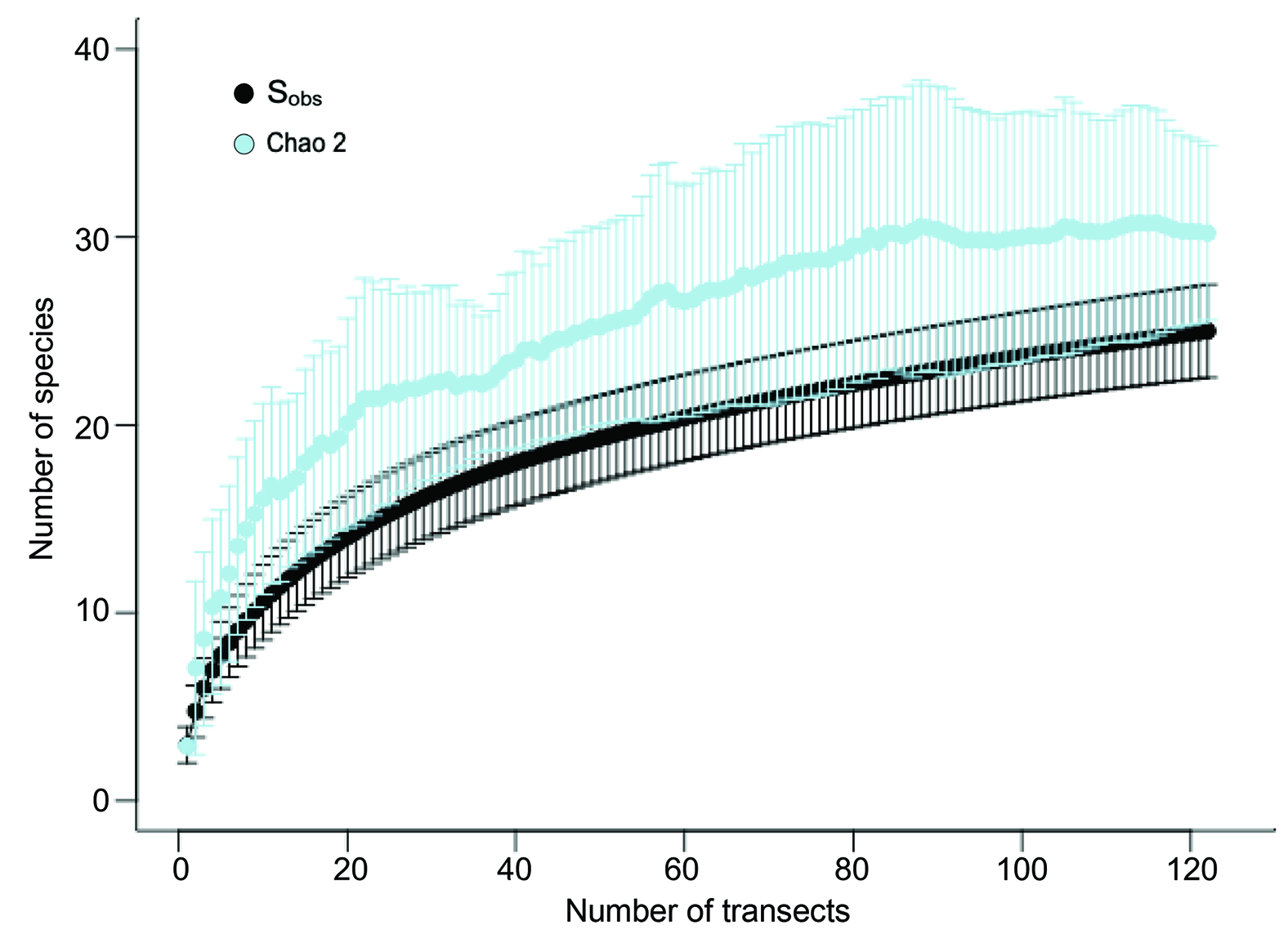

Supplement: S1 Fig — (TIF) [file pone.0257662.s001.tif]
