# Supplementary material for: Assemblage structure and spatial diversity patterns of kelp forest-associated fishes in Southern Patagonia
Source: PLoS One. 2021 Sep 20;16(9):e0257662. doi: 10.1371/journal.pone.0257662 (PMC8452001; doi:10.1371/journal.pone.0257662)
Supplement: S1 Table — Pisc = piscivore; Inv = invertivore. Size range in cm are from quantitative underwater transects. Family names in bold. *Magellanic endemic; + Magellanic, Subantarctic Is. endemic. Data on habitat are based on our personal observation and from previous work (Vanella et al. 2007; Fernández et al. 2012) (DOCX) [file pone.0257662.s002.docx]

**S1 Table**. Species observed during surveys in the Magellanic Province. Pisc = piscivore; Inv = invertivore. Size range in cm are from quantitative underwater transects. Family names in bold. *Magellanic endemic; ^+^ Magellanic, Subantarctic Is. endemic. Data on habitat are based on our personal observation and from previous work (Vanella et al. 2007; Fernández et al. 2012)

| Order | Family/  common name | Scientific name | Habitat | Trophic group | Size range |
| --- | --- | --- | --- | --- | --- |
| Myxiniformes | **Myxinidae**  Southern hagfish |  |  |  |  |
|  |  | *Myxine australis* | Benthic | Pisc, Inv | 15.0 – 30.0 |
| Gadiformes | **Muraenolepididae**  Eel cod  Eel cod |  |  |  |  |
|  |  | *Muraenolepis marmoratus*^+^ | Benthic | Pisc, Inv | 25.0 |
|  |  | *Muraenolepis orangiensis*^+^ | Benthic | Pisc, Inv | 25.0 |
| Ophidiiformes | **Ophidiidae**  Pink cuskeel |  |  |  |  |
|  |  | *Genypterus blacodes* | Benthic | Pisc, Inv | 38.0 |
| Scorpaeniformes | **Sebastidae**  Patagonian redfish |  |  |  |  |
|  |  | *Sebastes oculatus* | Benthic | Pisc, Inv | 24.0 |
|  | **Agonidae**  Armored fish |  |  |  |  |
|  |  | *Agonopsis chiloensis* | Benthic | Inv | 13.5 – 6.0 |
|  | **Liparidae**  Snailfish |  |  |  |  |
|  |  | *Careproctus pallidus** | Benthic | Inv | 10.0 |
| Syngnathiformes | **Syngnathidae** |  |  |  |  |
|  | Pipefish | *Leptonotus blainvilleanus* | Benthic | Inv | 15.0 – 25.0 |
| Perciformes | **Bovichtidae**  Frogmouth |  |  |  |  |
|  |  | *Cottoperca trigloides** | Benthic | Pisc, Inv | 12.0 – 35.0 |
|  | **Nototheniidae**  Magellanic rock cod  Rock cod  Rock cod  Rock cod  Rock cod  Rock cod  Rock cod  Rock cod |  |  |  |  |
|  |  | *Paranotothenia magellanica*+ | Semi-Pelagic | Inv | 4.5 – 20.0 |
|  |  | *Patagonotothen brevicauda** | Benthic | Inv | 5.0 – 16.5 |
|  |  | *Patagonotothen cornucola** | Benthic | Inv | 5.0 – 18.0 |
|  |  | *Patagonotothen longipes** | Benthic | Inv | 16.5 – 10.0 |
|  |  | *Patagonotothen sima** | Bentho-pelagic | Inv | 5.0 – 15.0 |
|  |  | *Patagonotothen squamiceps** | Benthic | Inv | 5.0 – 16.0 |
|  |  | *Patagonotothen tessellata** | Bentho-pelagic | Inv | 5.0 – 17.5 |
|  |  | *Patagonotothen* sp.* |  | Inv | 5.5 – 12.0 |
|  | **Harpagiferidae**  Spiny plunder fish |  |  |  |  |
|  |  | *Harpagifer bispinis** | Benthic | Inv | 4.5 – 8.0 |
|  | **Labrisomidae**  Labrisomid blenny |  |  |  |  |
|  |  | *Calliclinus geniguttatus* | Benthic | Pisc, Inv | 23.0 – 30.0 |
|  | **Tripterygiidae**  Triplefin |  |  |  |  |
|  |  | *Helcogrammoides cunninghami* | Benthic | Inv | 7.0 – 10.0 |
|  | **Zoarcidae**  S. American eelpout  Eelpout  Eelpout  Eelpout  Eelpout |  |  |  |  |
|  |  | *Austrolycus depressiceps** | Benthic | Pisc, Inv | 16.0 – 33.0 |
|  |  | *Crossostomus chilensis** | Benthic | Pisc, Inv | 10.0 – 18.0 |
|  |  | *Dadyanos insignis** | Benthic | Pisc, Inv | 15.0 |
|  |  | *Pogonolycus marinae** | Benthic | Pisc, Inv | 8.0 – 12.0 |
|  |  | *Piedrabuenia ringueleti** | Benthic | Pisc, Inv | 12.0 |
