# Supplementary material for: Assemblage structure and spatial diversity patterns of kelp forest-associated fishes in Southern Patagonia
Source: PLoS One. 2021 Sep 20;16(9):e0257662. doi: 10.1371/journal.pone.0257662 (PMC8452001; doi:10.1371/journal.pone.0257662)
Supplement: S2 Table — VIF: variance inflation factor; n/a: not assessed. (DOCX) [file pone.0257662.s003.docx]

**S2 Table**. Explanatory variables used in this study. VIF: variance inflation factor; n/a: not assessed.

| Variable | Type | VIF |
| --- | --- | --- |
| Exposure | Abiotic | n/a |
| Bottom type | Abiotic | n/a |
| Latitude (^o^) | Spatial | 4.82 |
| Longitude (^o^) | Spatial | 4.29 |
| Depth (m) | Abiotic | 2.33 |
| Temperature (^o^C) | Abiotic | 4.46 |
| Salinity (ppt) | Abiotic | 3.68 |
| Density of *M. pyrifera* (N^o^. m^-2^) | Biotic | 1.26 |
| Density of *Lessonia* spp. (N^o^. m^-2^) | Biotic | 1.56 |
