# Supplementary material for: Assemblage structure and spatial diversity patterns of kelp forest-associated fishes in Southern Patagonia
Source: PLoS One. 2021 Sep 20;16(9):e0257662. doi: 10.1371/journal.pone.0257662 (PMC8452001; doi:10.1371/journal.pone.0257662)
Supplement: S3 Table — edf: estimated degrees of freedom; AIC: Akaike information criterion; te: tensor product interaction; s: smooth term for predictor variables. P < 0.05 is indicated in bold. (DOCX) [file pone.0257662.s004.docx]

**S3 Table**. Best fitting generalized additive models for the total number of fish species, Shannon-Weiner diversity, Numerical abundance and biomass. edf: estimated degrees of freedom; AIC: Akaike information criterion; te: tensor product interaction; s: smooth term for predictor variables. *P* < 0.05 is indicated in bold.

| Response variable | Best-fitting model | edf | Deviance explained | | | AIC | *P*-value |
| --- | --- | --- | --- | --- | --- | --- | --- |
| Total number of fish species (*S*) | Exposure + bottom type + te(Lat, Lon) + s(Temp) + s(Depth) + s(*Lessonia*_density) | 3.00, 3.910, 1.00, 2.01 | | 41.9% | 389.11 | | 0.09, **0.01**, 0.15, **0.03** |
|  |  |  | |  |  | |  |
| Shannon-Weiner diversity (*H’*) | Exposure + bottom type + te(Lat, Lon) + s(Temp) + s(Depth) + s(*Lessonia*_density) | 3.00, 3.926, 1.00, 2.478 | | 44.0% | 136.65 | | **0.01, 0.01, 0.03, <0.01** |
|  |  |  | |  |  | |  |
| Numerical abundance  (Num m^-2^) | Exposure + bottom type + s(Lat) + s(Temp) + s(Depth) + s(*Lessonia*_density) | 5.07, 2.33, 1.00, 2.891 | | 57.5% | 67.78 | | **<0.001,** 0.07**, 0.01, <0.01** |
|  |  |  | |  |  | |  |
| Biomass (g m^-2^) | Exposure + bottom type + te(Lat, Lon) + s(Depth) + s(*Lessonia*_density) | 5.30, 1.49, 2.35 | | 37.6% | 777.28 | | 0.18, **0.02, 0.01** |
